# Supplementary material for: Short Tandem Repeats (STRs) as Biomarkers for the Quantitative Follow-Up of Chimerism after Stem Cell Transplantation: Methodological Considerations and Clinical Application
Source: Genes (Basel). 2020 Aug 25;11(9):993. doi: 10.3390/genes11090993 (PMC7565503; doi:10.3390/genes11090993)
Supplement: Supplementary file 1 [file genes-11-00993-s001.pdf]

Supplementary Information

STR polymorphisms used for post-stem cell transplant chimerism quantification.

The commercial kit used in this study (Mentype®Chimera®, Biotype GmbH, Germany) include highly polymorphic short tandem repeats (STRs) with a very high rate of heterozygosity and a balanced allelic distribution (Thiede et al. 2004). Specifically, one PCR reaction simultaneously amplifies the autosomal loci D2S1360, D3S1744, D4S2366, D5S2500, D6S474, D7S1517, D8S1132, D10S2325, D12S391, D18S51, D21S2055, SE33 (ACTBP2), and the gender-specific locus Amelogenin (detailed in Supplementary Table S1).

Locus	GenBank accession	Repeat motif of the reference allele	Reference allele	Allele range	Chromosomal Mapping
D2S1360	G08130	$[TATC]_9$ $[TGTC]_9$ $[TATC]_5$	23	19-32	2p24-p22
D3S1744	G08246	$[TCTA]_2$ TA$[TCTA]_{12}$ TCA $[TCTA]_2$	16	13-22	3p24
D4S2366	G08339	$[ATAG]_9$ ATTG $[ATAG]_2$	12	9-15	4p16-15.2
D5S2500	G08468	[ATAG]12	12	9-18	5q11.2
D6S474	G08540	$[TAGA]_5$ TGA $[TAGA]_{12}$	17	11-20	6q21-22
D7S1517	G18365	$[GAAA]_{11}$ CAAA $[GAAA]_2$ CAAA $[GAAA]_2$	17	14-31	7q31.33
D8S1132	G08685	$[TCTA]_9$ TCA $[TCTA]_9$ TCTGTCTA	20	12.1-27	8q23.1
D10S2325	G08790	[TCTTA]12	12	6-23	10p12
D12S391	G08921	$[AGAT]_5$ GAT $[AGAT]_7$ $[AGAC]_6$ AGAT	19.3	13-28	12p13.2
D18S51	L18333	[AGAA]13	13	5.3-42	18q21.3
D21S2055	G27274	$[CTAT]_2$ CTAA $[CTAT]_9$ CTA $[CTAT]_3$ TAT $[CTAT]_3$ TAT $[CTAT]_4$ CAT$[CTAT]_2$	24	16.1-39	21q22
SE33 (ACTBP2)	NG000840	$[AAAG]_9$ AA $[AAAG]_{16}$	25.2	3-50	6q14.2
Amelogenin X	M55418				Xp22.1-22.3
Amelogenin Y	M55419				Yp11.2

Supplementary Table S1.- STR loci used for STR-PCR analysis. Detailed information on the STR loci used for chimerism quantification in the present study. For further information refer to the Instructions for Use of Mentype®Chimera® (available on www.biotype.de).

References

Thiede C, Bornhäuser M and Ehninger G. Evaluation of STR informativity for chimerism testing – comparative analysis of 27 STR systems in 203 matched related donor recipient pairs. *Leukemia.* **2004**, 18, 248–254. doi:10.1038/sj.leu.2403212.

Supplementary Table S2.

BM		qPCR +	qPCR -
STR-PCR	+	9	0
STR-PCR	-	57	16

PB		qPCR +	qPCR -
STR-PCR	+	12	0
STR-PCR	-	15	40

Supplementary Table S2.- Concordance of positive and negative results by STR-PCR and qPCR in PB and in BM samples. Positive (presence of recipient DNA, MC) and negative (absence of recipient DNA, CC) results by STR-PCR and qPCR in BM (left panel, n=82), and PB (right panel, n=67) samples. Of note, the proportion of positive samples by qPCR that are negative by STR-PCR is higher in BM (57/82, 70%) than in PB samples (15/67, 22%).

Supplementary Figure S1.

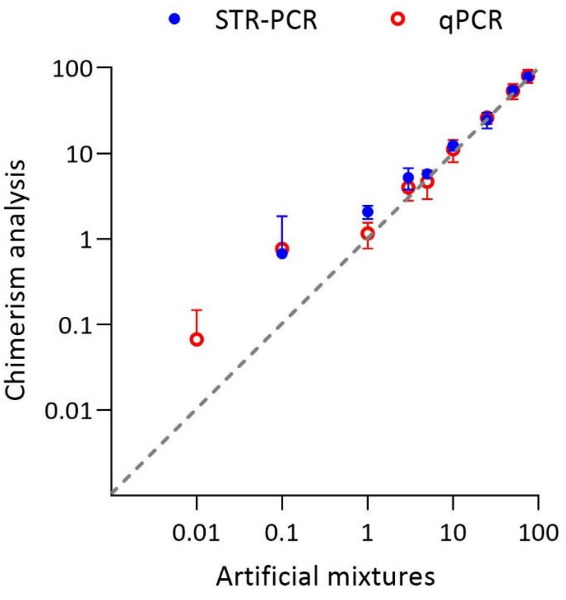

Supplementary Figure S1.- Artificial mixture analysis represented in a logarithmic scale. (A) Results of chimerism quantification using STR-PCR (blue) and qPCR (red), vs. actual percentage of cells of putative recipient (male) are represented using a logarithmic scale.
